# Carbon-Based Fe_3_O_4_ Nanocomposites Derived from Waste Pomelo Peels for Magnetic Solid-Phase Extraction of 11 Triazole Fungicides in Fruit Samples

**DOI:** 10.3390/nano8050302

**Published:** 2018-05-06

**Authors:** Keyu Ren, Wenlin Zhang, Shurui Cao, Guomin Wang, Zhiqin Zhou

**Affiliations:** 1 College of Horticulture and Landscape Architecture, Southwest University, Chongqing 400716, China; renkeyu1010@163.com; 2 Chongqing Key Laboratory of Economic Plant Biotechnology, Collaborative Innovation Center of Special Plant Industry in Chongqing, Institute of Special Plants, Chongqing University of Arts and Sciences, Yongchuan 402160, China; zhangwenlin88519@126.com; 3 The Inspection Technical Center of Chongqing Entry-Exit Inspection & Quarantine Bureau, Chongqing 400020, China; chqwgm@163.com; 4 Key Laboratory of Horticulture Science for Southern Mountainous Regions, Ministry of Education, Chongqing 400715, China

**Keywords:** carbon based Fe_3_O_4_ nanocomposites, pomelo peels, magnetic solid phase extraction, triazole fungicides

## Abstract

Carbon-based Fe_3_O_4_ nanocomposites (C/Fe_3_O_4_ NCs) were synthesized by a simple one-step hydrothermal method using waste pomelo peels as the carbon precursors. The characterization results showed that they had good structures and physicochemical properties. The prepared C/Fe_3_O_4_ NCs could be applied as excellent and recyclable adsorbents for magnetic solid phase extraction (MSPE) of 11 triazole fungicides in fruit samples. In the MSPE procedure, several parameters including the amount of adsorbents, extraction time, the type and volume of desorption solvent, and desorption time were optimized in detail. Under the optimized conditions, the good linearity (*R*^2^ > 0.9916), the limits of detection (LOD), and quantification (LOQ) were obtained in the range of 1–100, 0.12–0.55, and 0.39–1.85 μg/kg for 11 pesticides, respectively. Lastly, the proposed MSPE method was successfully applied to analyze triazole fungicides in real apple, pear, orange, peach, and banana samples with recoveries in the range of 82.1% to 109.9% and relative standard deviations (RSDs) below 8.4%. Therefore, the C/Fe_3_O_4_ NCs based MSPE method has a great potential for isolating and pre-concentrating trace levels of triazole fungicides in fruits.

## 1. Introduction

Triazole fungicides, which are typically comprised of a 1,2,4-triazole moiety, a hydroxy (keto) group, and substituted benzyl [1], have been employed as systemic fungicides because of their high capability to hinder the biosynthesis of steroid hormones [2]. However, the improper use of these compounds has resulted in undesirable residues on fruits, which increase the risk of transferring the residual pesticides from the skin of fruits to consumers’ body. More importantly, triazole fungicides in fruits can potentially lead to endocrine-related side effects, hepatotoxicity, and teratogenic effects on humans [3]. Therefore, it is necessary to detect and clear their contents in fruits. Due to the trace level concentrations of these compounds, an efficient sample preparation technique before detection is essential prior to instrumental measurement directly to obtain the reliable results.

Several sample preparation techniques have been developed to extract and pre-concentrate triazole fungicides such as liquid-liquid extraction (LLE), solid-phase extraction (SPE), solid-phase microextraction (SPME), and dispersive liquid-liquid microextraction (DLLME) [4]. Among them, liquid-liquid extraction (LLE) and solid phase extraction (SPE) are the most common methods. Nevertheless, they are still tedious, time consuming, and relatively expensive. Recently, SPE-based magnetic solid phase extraction (MSPE) has drawn more attention due to its advantages of high efficiency, low cost, and environmental friendliness. In MSPE, the magnetic adsorbent is directly added to a sample solution containing the target compounds and is easily separated by an external magnetic field instead of filtration or the centrifugation process, which make the separation become easier and faster [5]. Moreover, the adsorbent used in MSPE play a key role in efficient extraction. Lately, carbon-based magnetic nanomaterials including carbon nanotubes [6], graphene [7], metal-organic framework derived carbon [8], and activated carbon [9] were applied as adsorbents for MSPE due to their high adsorption ability, easy separation, and better performance in sample preparation. Recently, various natural biomass such as corn stalk [10], peanut shells [11], and more were employed to fabricate carbon-based magnetic materials owing to their low price, wide source, high efficiency, and friendly-environment. These prepared materials could be used as adsorbents for extraction of carbamates pesticides or phenylurea herbicides in river water and rose juice sample. 

Pomelo, one of the characteristic fruits in China, is consumed in large amounts every year. The pomelo peels (PPs) account for 44% to 54% of the fresh fruit, which serve little economic purpose. Noticeably, a huge amount of PPs is usually discarded as waste, which leads to environmental problems. However, PPs contain rich plant fiber and many functional groups such as hydroxyl, carboxyl, and amidogen make it become a promising adsorbent [12]. It has been reported that PPs were employed to fabricate carbon-based materials for waste water treatment [13], heavy metal determination [14], and super-capacitor applications [15]. To our knowledge, unique carbon-based Fe_3_O_4_ nanomaterials from PPs as adsorbents for MSPE has not yet been reported. 

**Figure 1:**
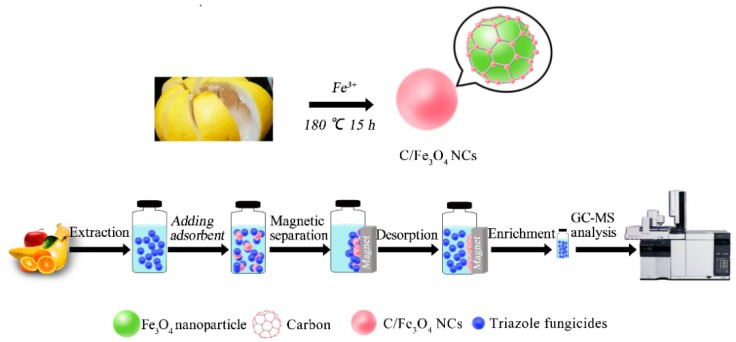
Illustration of the procedure for synthesis of C/Fe_3_O_4_ NCs and MSPE steps for triazole fungicides analysis in fruits.

In this study, we employed waste PPs to prepare the carbon-based Fe_3_O_4_ nanocomposites (C/Fe_3_O_4_ NCs) by a simple one-step hydrothermal method (see Figure 1). Subsequently, the prepared C/Fe_3_O_4_ NCs were used as adsorbents for extracting 11 triazole fungicides from apple, pear, orange, and banana samples (see Figure 1). Lastly, the C/Fe_3_O_4_ NCs-based MSPE method was proposed, which has a great potential for isolating and pre-concentrating trace levels of triazole fungicides in fruits.

## 2. Results and Discussion

### 2.1. Characterization of C/Fe_3_O_4_ NCs

**Figure 2:**
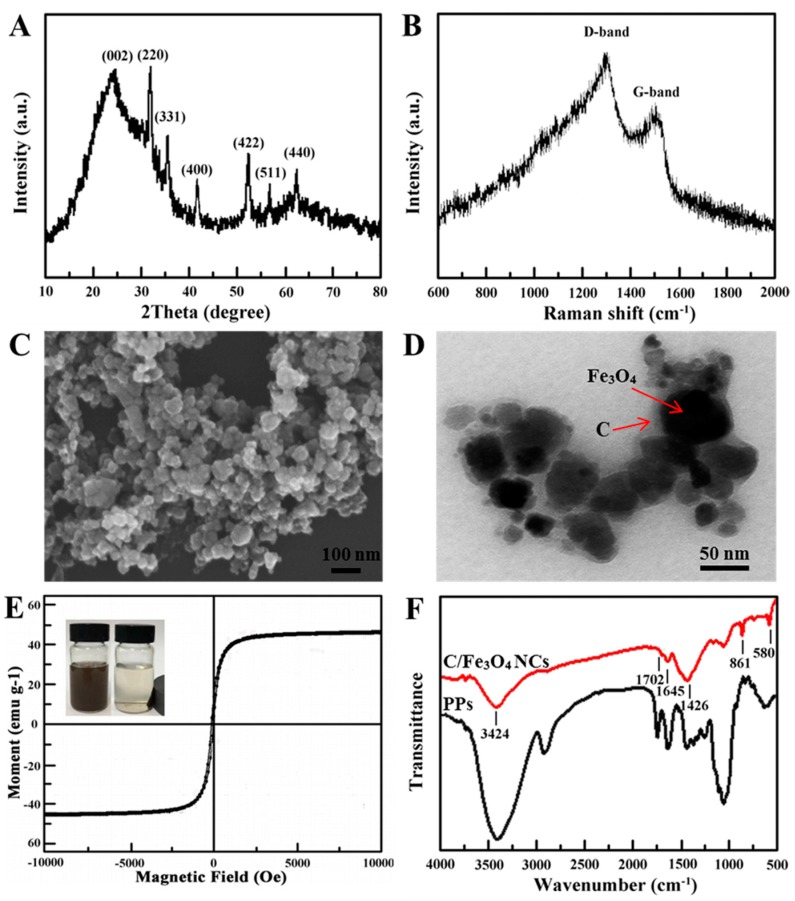
(**A**) X-ray diffraction pattern, (**B**) Raman spectrum, (**C**) SEM image, (**D**) TEM image, (**E**) VSM magnetization curve of C/Fe_3_O_4_ NCs, and (**F**) FT-IR spectra of C/Fe_3_O_4_ NCs and PPs.

First, the crystalline structure of C/Fe_3_O_4_ NCs was investigated by using XRD. As seen in Figure 2A, the broaden peaks of C/Fe_3_O_4_ NCs at 2θ = 25.8° were attributed to amorphous carbon. The diffraction peaks at 2θ = 30.3°, 35.5°, 43.3°, 53.2°, 57.2°, and 62.8° corresponded to (220), (311), (400), (422), (511), and (440) facets of Fe_3_O_4_ [16], respectively, which indicates that Fe_3_O_4_ nanoparticles were successfully synthesized using a face-centered cubic structure. The raman spectrum indicated that the peaks at 1357 cm^−1^ (D-band) and 1590 cm^−1^ (G-band) (see Figure 2B) were associated with sp^3^ and sp^2^ hybridized carbon [16]. Subsequently, the morphology of C/Fe_3_O_4_ NCs was observed by SEM and TEM. As shown in Figure 2C, C/Fe_3_O_4_ NCs were approximately spherical with the average size of about 50 nm. In Figure 2D, the carbon could be observed as a light area surrounding the dark core of Fe_3_O_4_, which suggested that the carbon derived from pomelo peels was successfully incorporated with Fe_3_O_4_ nanoparticles to form C/Fe_3_O_4_ NCs. The carbon shell endowed C/Fe_3_O_4_ NCs with strong adsorption ability. Additionally, the prepared C/Fe_3_O_4_ NCs had superparamagnetic behavior with a high saturation magnetization of 45.9 emu g^−1^ at room temperature (see Figure 2E). At the same time, C/Fe_3_O_4_ NCs could be easily dispersed in water and separated by an external magnetic field. Finally, the surface groups of C/Fe_3_O_4_ NCs was studied by Fourier transform-infrared spectroscopy(FT-IR). Figure 2F represented the FT-IR spectra of PPs and C/Fe_3_O_4_ NCs. As can be observed, the peak at 580 cm^−1^ ascribed to the vibration of Fe–O bond on C/Fe_3_O_4_ NCs. The bands at 3424 cm^−1^, 2930 cm^−1^, 1702 cm^−1^, and 1645 cm^−1^ corresponded to O–H, C–H, C=O, and C=C, respectively, which were attributed to the carbonization of PPs during the hydrothermal process [17]. 1000 cm^−1^ to 1460 cm^−1^ can be associated with C–O stretching vibrations in acids, alcohols, phenols, ethers, esters, and O–H bending vibrations, which suggests the presence of a large amount of hydrophilic groups [18,19]. The bands at 700 cm^−1^ to 900 cm^−1^ were assigned to the C–H out-of-plane bonding in benzene derivatives [20], which might have adsorbed some benzenoid compounds by using π-π interaction. Based on the above results, there were rich oxygen-containing groups on C/Fe_3_O_4_ NCs surface, which made C/Fe_3_O_4_ NCs disperse well in solution for practical application.

### 2.2. MSPE Optimization

#### 2.2.1. Effect of Activation Factor

**Figure 3:**
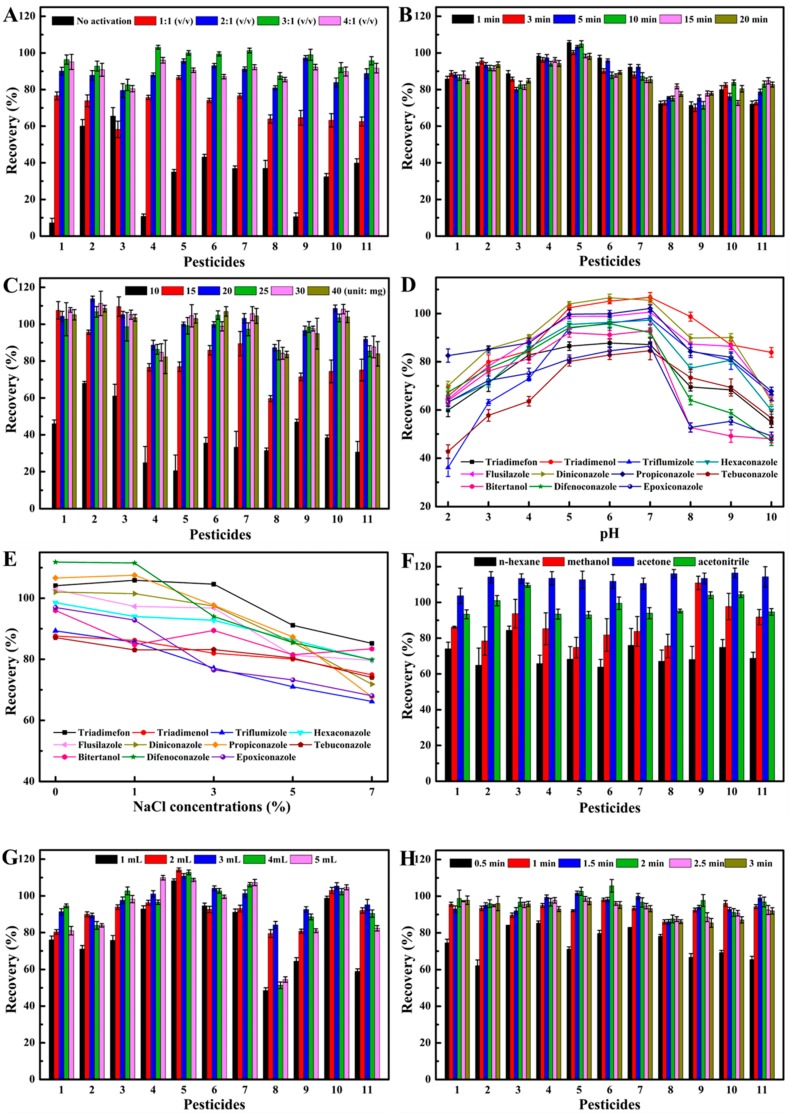
Effects of (**A**) ratio of activation, (**B**) extraction time, (**C**) amount of adsorbent, (**D**) pH of extraction solvent, (**E**) salt concentration, (**F**) type of desorption solvent, (**G**) volume of desorption solvent, (**H**) and desorption time on the MSPE performance. (1. Triadimefon, 2. Triadimenol, 3. Triflumizole, 4. Hexaconazole, 5. Flusilazole, 6. Diniconazole, 7. Epoxiconazole, 8. Propiconazole, 9. Tebuconazole, 10. Bitertanol, 11. Difenoconazole).

In this work, the hydrosolvent and organic solvents were tested to select the most proper extractant. The results indicated that the hydrosolvent is the best choice. Additionally, the activation of C/Fe_3_O_4_ NCs was carried out to improve the recoveries of pesticides. As shown in Figure 3A, the highest extraction efficiencies were obtained using acetonitrile/toluene (3:1, *v*/*v*), which was used to activate the adsorbent. The highest recoveries were attributed to the addition of toluene by avoiding the irreversible adsorption of target analytes [21]. Therefore, the process of activating materials was indispensable for improving the extraction efficiency. 

#### 2.2.2. Effect of Extraction Time and Adsorbent Amount

Generally, extraction time is a significant factor for achieving the adsorption equilibrium between the analytes and the adsorbents. Figure 3B showed that the recoveries of triazole fungicides had no obvious fluctuation when the shaking time was changed from 1 min to 20 min, which indicates that rapid equilibrium occurred before the first minute. C/Fe_3_O_4_ NCs could be uniformly dispersed into the extraction solution by using the platform shaker, which makes a large contact surface area between the adsorbent molecules and the fungicide molecules for a fast mass transfer [18]. Therefore, the shaking time of 1 min was selected as the optimal extraction time. To achieve the high extraction recovery of the analytes, the dosage of C/Fe_3_O_4_ NCs was investigated, which ranged from 5 mg to 30 mg. As shown in Figure 3C, the recoveries for 11 triazole fungicides increased as the amount of adsorbent rose to 20 mg and then remained almost invariant when the amount of the adsorbent grew further. Therefore, 20 mg of the adsorbent was used in the following studies.

#### 2.2.3. Effect of pH 

The pH of the sample solution is an important parameter that influences the characteristics of adsorbent and existing forms of analytes. Therefore, the effect of solution pH on the triazole fungicides extraction recoveries was investigated by adjusting pH from 3 to 10 by HCl or NaOH. Figure 3D revealed that the extraction recoveries of 11 triazole fungicides had no significant change when pH was changed from 5 to 7. However, they clearly decreased when pH was lower than 5 or higher than 8 due to the degradation of fungicides during these conditions. Additionally, the oxygen-groups on an adsorbent surface were ionized at alkaline conditions and adsorbed more water molecules, which hindered the triazole fungicides molecules into the adsorption sites of C/Fe_3_O_4_ NCs and resulted in the decrease of extraction recoveries [22]. Since the pH of the sample solution was 5.5–6.5, there was no need to adjust the pH of the extraction solution.

#### 2.2.4. Effect of Salinity

The effect of salt concentrations on the extraction recoveries of triazole fungicides using C/Fe_3_O_4_ NCs was explored by adding different amounts of NaCl ranging from 0% to 7% (*w*/*v*). As illustrated in Figure 3E, the extraction recoveries for all triazole fungicides were decreased with the growth of NaCl concentrations. It was due to the fact that the salt could decrease the solubility of analytes, which blocked the mass transfer of analytes from solution to adsorbent. Furthermore, Na^+^ might occupy some adsorption sites of C/Fe_3_O_4_ NCs surface, which leads to the decrease of extraction efficiency. Therefore, no salt was added to the extraction solvent in the subsequent experiments.

#### 2.2.5. Effect of Desorption Agent Type

An appropriate desorption solvent is crucial for improving desorption efficiency. As such, different organic solvents including methanol, *n*-hexane, acetone, and acetonitrile were tested to select the most suitable desorption solvent in MSPE. As shown in Figure 3F, acetonitrile and acetone had higher extraction efficiency in comparison with other solvents. Since acetone could dissolve some impurities in complex sample matrices, acetonitrile was superior to acetone for desorption of analytes. Particularly, the lowest recoveries obtained from *n*-hexane could be due to the weaker dispersibility of the adsorbent in the solvent, which leads to the agglomeration of adsorbent and prevents the effective desorption of analytes [22]. According to the above results, acetonitrile was chosen as the best desorption solvent.

#### 2.2.6. Effect of Desorption Solvent Volume and Desorption Time

The effect of desorption solvent volume on the extraction recovery of analytes was investigated. In a series of optimization experiments, 1 mL to 5 mL of acetonitrile was used to elute the analytes. As seen in Figure 3G, 3 mL of acetonitrile was sufficient to elute triazole fungicides from C/Fe_3_O_4_ NCs completely. Moreover, the desorption time was studied by increasing the vortex duration from 0.5 min to 3 min. Figure 3H showed that no significant changes were observed for the extraction recoveries of 11 triazole fungicides after 1 min. Therefore, the vortex time of 1 min was selected to complete desorption of analytes from the adsorbent.

### 2.3. Validation of the Method

The validation of the developed MSPE gas chromatography-mass spectrometer (MSPE-GC-MS) method for analyzing triazole fungicides was evaluated under the optimized experimental conditions. Effective quality assurance and quality control (QA/QC) measures were carried out for monitoring triazole fungicides. The quantitative parameters including linearity, limit of detection (LOD), limit of quantification (LOQ), repeatability, and reproducibility were determined to validate the MSPE-GC-MS method.

A series of blank water samples and fruit samples spiked with triazole fungicide standards at different concentration levels were prepared to establish the standard and matrix-matched calibration curves. For each level, three replicate extraction and determinations were performed and the calibration curve of each triazole fungicide was plotted to target the quantitative ion peak area *y* versus the corresponding concentration of the analytes *x*. Matrix effects (ME) were evaluated by the slope ratio of the calibration curves (solvent standard calibration and matrix-matched calibration) for 11 triazole fungicides in four different matrices. The results (see Table S1) showed that there was no significant difference, which indicates that ME could be ignored. However, the matrix-matched calibration was used for an accurate quantification and the analytical results are shown in Table 1. The good linearity was achieved in the concentration range of 1 μg/kg to 100 μg/kg with satisfactory correlation coefficients (*R*^2^ > 0.9916). The LOD and LOQ of the method were found in the range of 0.12 μg/kg to 0.55 μg/kg and 0.39 μg/kg to 1.85 μg/kg, which were calculated based on the signal to noise ratio of 3 (*S*/*N* = 3) and 10 (*S*/*N* = 10), respectively. Moreover, the repeatability and reproducibility of the method were also investigated by intra-day and inter-day precisions. As shown in Table 2, the recoveries, intra-day relative standard deviations (RSDs), and inter-day relative standard deviations (RSDs) of 11 triazole fungicides in spiked samples were in the range of 82.1% to 109.9%, 2.1% to 6.6%, and 3.5% to 8.4%, respectively, which indicated that our developed analytical method had high sensitivity and good repeatability.

Additionally, in order to evaluate the analytical protocols relating to green analytical chemistry, the Analytical Eco-Scale [23] and Green Analytical Procedure Index (GAPI) [24] tools should be employed. Analytical Eco-Scale compares different steps and parameters in an analytical process, but it does not give comprehensive information of evaluated protocols. However, GAPI could provide more supplemental information on the whole procedure from sample preparation to determination. Therefore, we can use GAPI to evaluate the MSPE-GC-MS analytical procedure in detail for further study.

### 2.4. Reusability of C/Fe_3_O_4_ NCs

**Figure 4:**
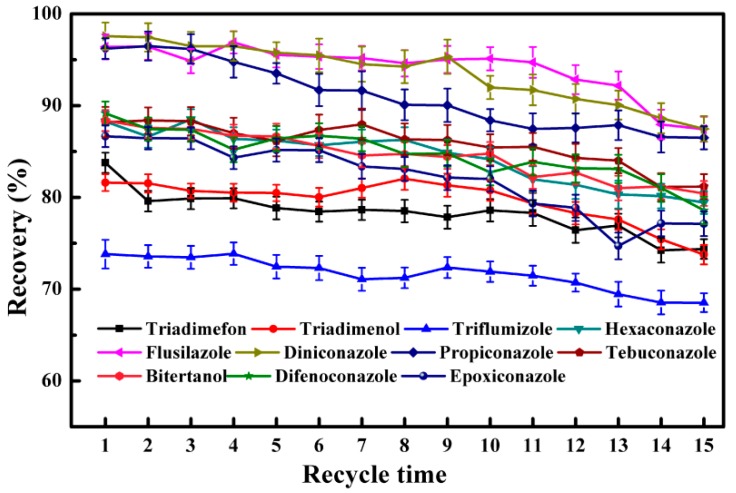
Effect of recycle times on the recoveries of 11 triazole fungicides.

In order to investigate the reusability of C/Fe_3_O_4_ NCs, the used adsorbent was washed twice with 3 mL of acetonitrile before the next MSPE procedure. As shown in Figure 4 and Table S2, the recoveries of 11 triazole fungicides are significant differences (*p* < 0.05) between the 15th cycle and the first cycle. However, the decrease of recovery is less than 10%, which indicates that C/Fe_3_O_4_ NCs could be recycled. This was in accordance with other reports [22,25]. Therefore, C/Fe_3_O_4_ NCs had great potential for recycling in the sample preparation.

### 2.5. Analysis of Real Samples

Furthermore, the performance of optimization MSPE method was evaluated by different fruit samples including apples, pears, oranges, peaches, and bananas. The 11 triazole fungicide residuals were presented in Table 3. The results showed that there were 0.3 μg/kg to 0.5 μg/kg hexaconazole observed in apples, pears, and peaches, 0.2 μg/kg and 0.3 μg/kg flusilazole in apples and peaches, 0.2 μg/kg bitertanol in apples, and 0.4 μg/kg triadimefon in pears, respectively. According to our results, common dietary consumption of fruits is safe since the amount of triazole fungicides residue in these fruits were significantly lower than maximum residue limits (MRLs).

### 2.6. Comparison with Other Methods

Finally, the proposed method was compared with other previously reported methods by the determination of triazole fungicides. As listed in Table 4, the MSPE-GC-MS method based on C/Fe_3_O_4_ NCs could be used for analyzing multiple analytes and had short extraction time, lower RSD%, and comparable LOD when compared with previous reports. The prepared adsorbent derived from pomelo peels was cheap and could reduce the resources waste and environment pollution caused by pomelo peels. Therefore, this method had the advantages of high accuracy, sensitivity, and rapidity as well as being low-cost and eco-friendly. 

## 3. Materials and Methods

### 3.1. Materials

Preservative standards (Table 1, analytic grade, and purity ≥ 99.0%) were bought from Dr. Ehrenstorfer GmbH. (Augsburg, Germany). Urea (U5378, powder) was purchased from Sigma-Aldrich Co. LLC. (St Louis, MI, USA). Ferric chloride hexahydrate (FeCl_3_·6H_2_O, AR, 99.0%) and other chemicals were all purchased from Chengdu Kelong Chemical Reagent Co. (Chengdu, China). Acetonitrile, acetone, *n*-hexane, and methanol were purchased from Tedia (Fairfield, CT, USA). Double-distilled water was prepared by Milli-Q-plus ultra-pure water system (Milford, MA, USA) throughout the work. The pomelo peel and apple, pear, orange, peach, and banana were obtained from local supermarkets (Chongqing, China).

### 3.2. Instruments

Chromatographic analyses were performed with an Agilent GC-MS with 7890B GC connected to an Agilent 5977A Triple-Axis mass detector (Agilent, Santa Clara, CA, USA). The separation of column was HP-5 ms capillary column (30 m × 0.25 mm × 0.25μm, Agilent). The instrument was equipped with a splitless injector and the carrier gas was ultrapure helium (purity ≥ 99.999%) with a flow rate of 1.0 mL min^−1^. The oven temperature was programmed from 80 °C for 2 min to 180 °C for 5 min at the rate of 20 °C min^−1^. Then the temperature was raised to 280 °C at the rate of 10 °C min^−1^ and finally went up to 290 °C with 5 °C min^−1^. The temperatures of interface, ion source, injection port, and quadrupole were held at 280 °C, 230 °C, 250 °C, and 150 °C, respectively. The selective ion monitoring (SIM) mode was adopted for the quantitative analysis. The volume of splitless injection was 1 μL. The information of qualitative and quantitative ions for compounds and the typical retention time were listed in Table 1. The morphology of prepared C/Fe_3_O_4_ NCs was observed by a JSM-6510LV scanning electron microscope (SEM, Tokyo, Japan) and a Tecnai G2F20 S-Twin transmission electron microscope (TEM, Hillsboro, OR, USA). X-ray diffraction (XRD) patterns were performed using a Shimadzu XRD-7000 diffractometer (Kyoto, Japan). The FT-IR spectra were obtained on Tensor 37 (Bruker Spectrometer Company, Ettlingen, Germany). Magnetic hysteresis loops were studied using a Lake Shore 7303 model vibrating sample magnetometer (VSM) (Lake Shore, WA, USA) in a magnetic field range from −10,000 Oe to 10,000 Oe at 25 °C.

### 3.3. Synthesis of C/Fe_3_O_4_ NCs 

C/Fe_3_O_4_ NCs were synthesized using waste sponge-like pomelo peels (see Figure 1) by a one-step hydrothermal method. The pomelo peel (0.1 g), FeCl_3_·6H_2_O (0.1 g), and urea (0.3 g) were added to 15 mL of ultrapure water under vigorous stirring for 30 min. Subsequently, the mixture was transferred into a 25 mL Teflon-lined stainless steel autoclave for hydrothermal carbonization at 180 °C for 15 h. After the autoclave cooled down to room temperature, the black products were separated by a magnet, washed with ethanol and ultrapure water, and freeze-dried under vacuum.

### 3.4. Sample Preparation and MSPE Procedure 

The homogenate of fresh fruits including apples, pears, oranges, and bananas were obtained by a laboratory homogenizer. 10.0 g of the homogenized sample was placed into a 50 mL Teflon centrifuge tube. Afterward, 20 mL ACN and 5.0 g NaCl were added. The mixture was shaken for 15 min and then centrifuged at 5000 rpm for 2 min. The collected supernatant was evaporated to dryness and re-dissolved using 5 mL of water. The schematic MSPE procedure was shown in Figure 1. First, 20 mg of C/Fe_3_O_4_ NCs was activated by 4 mL acetonitrile-toluene solution (3:1, *v*/*v*) by shaking for 5 min at medium speed with a platform shaker. Next, the activated adsorbent was added to the above aqueous solution and shaken for 1 min. Subsequently, the adsorbent was isolated from the water phase by a magnet. In the next step, the pre-concentrated target analytes were eluted from the adsorbent with 3 mL acetonitrile by vigorous vortex for 1 min. The desorbed solutions were evaporated to dryness. Lastly, the residue was re-dissolved in 1 mL of acetone and 1 μL of it was taken for GC-MS analysis. 

## 4. Conclusions

Novel C/Fe_3_O_4_ NCs, which were used as recyclable adsorbents for MSPE of 11 triazole fungicides in fruit samples, were successfully synthesized by a simple one-step hydrothermal approach using waste pomelo peels as carbon precursors. The prepared C/Fe_3_O_4_ NCs not only had the fast magnetic separation of Fe_3_O_4_ nanoparticles but also had the high extraction efficiency of carbon for target analytes. Furthermore, the C/Fe_3_O_4_ NCs-based MSPE-GC-MS method performed good linearity, high recovery, low LOD, and satisfactory RSD, which indicated that it was sensitive and accurate for triazole fungicide analysis in fruit samples. Therefore, the proposed C/Fe_3_O_4_ NCs-based MSPE method has a great potential application for testing institutions to isolate and pre-concentrate trace levels of triazole fungicides in fruits.

## Figures and Tables

**Table 1:** The retention times, target ions, and analytical parameters of the MSPE-GC-MS method for 11 triazole fungicide compounds.

Compounds	Rt (min)	Quantifier and Qualifier (*m*/*z*)	Regression Equations	*R* ^2^	LOD (μg/kg)	LOQ (μg/kg)
Triadimefon	15.395	**208**, 81, 210	*y* = 30.26*x* + 1262	0.9916	0.15	0.50
Triadimenol	16.586	**112**, 168, 130,	*y* = 34.65*x* + 2031	0.9990	0.26	0.88
Triflumizole	16.785	**206**, 179, 186	*y* = 13.99*x* + 464	0.9997	0.32	1.08
Hexaconazole	17.467	**214**, 231, 256	*y* = 17.15*x* + 907.2	0.9951	0.26	0.86
Flusilazole	18.005	**233**, 315, 206	*y* = 89.79*x* + 2170	0.9970	0.12	0.39
Diniconazole	18.600	**268**, 270, 232	*y* = 39.38*x* − 527.5	0.9947	0.14	0.46
Epoxiconazole	19.395 20.019	**192**, 183, 138	*y* = 19.39*x* + 340.5	0.9930	0.55	1.85
Propiconazole	19.403 19.539	**259**, 171, 261	*y* = 42.2*x* + 413.4	0.9999	0.13	0.45
Tebuconazole	19.866	**250**, 252, 163	*y* = 31.35*x* + 474	0.9984	0.16	0.54
Bitertanol	22.406	**170**, 112, 141	*y* = 76.21*x* + 1352	0.9951	0.18	0.58
Difenoconazole	25.309	**323**, 325, 265	*y* = 29.65*x* + 779.4	0.9982	0.15	0.51

*y*: peak area; *x*: mass concentration, μg/L. Linear range: 1 μg/kg to 100 μg/kg. The bold represents quantitation ions of 11 triazole fungicides.

**Table 2:** The precision of MSPE-GC-MS method for 11 triazole fungicides.

Compounds	Spiked Level (μg/kg)	Intra-Day (*n* = 6)		Inter-Day (*n* = 6)	
		Recovery (%)	RSD (%)	Recovery (%)	RSD (%)
Triadimefon	10	87.6	5.4	85.6	6.7
	20	91.2	3.6	89.6	4.4
	50	96.8	2.3	93.3	4.6
Triadimenol	10	90.6	4.2	87.7	5.9
	20	92.5	4.1	88.6	4.7
	50	104.3	2.6	97.1	3.5
Triflumizole	10	109.9	5.2	102.8	6.5
	20	104.2	3.2	97.2	6.1
	50	97.8	2.1	98.1	3.8
Hexaconazole	10	98.2	4.9	95.1	7.6
	20	99.2	3.2	95.9	4.9
	50	103.5	2.6	97.7	3.8
Flusilazole	10	108.7	6.6	104.0	8.4
	20	98.5	4.2	95.3	6.4
	50	99.2	3.2	96.6	4.5
Diniconazole	10	103.1	4.7	96.4	7.9
	20	101.0	3.8	98.5	4.6
	50	102.4	2.8	97.7	3.7
Epoxiconazole	10	103.8	5.4	98.4	5.7
	20	99.1	3.9	96.9	4.8
	50	101.5	3.2	99.4	3.6
Propiconazole	10	85.7	4.6	83.5	6.0
	20	92.5	4.3	86.1	5.6
	50	95.4	3.6	89.4	4.2
Tebuconazole	10	83.9	6.1	82.1	6.9
	20	90.0	5.2	86.1	7.0
	50	96.3	3.4	95.0	4.2
Bitertanol	10	107.8	6.2	98.1	7.3
	20	96.2	5.1	94.3	5.4
	50	102.1	2.5	100.7	4.6
Difenoconazole	10	89.0	5.0	84.6	5.3
	20	92.6	4.2	90.3	4.8
	50	93.9	2.9	92.3	4.2

**Table 3:** 11 triazole fungicide residues in real samples (μg/kg).

Compounds	Apple	Pear	Orange	Banana	Peach
Triadimefon	ND	0.41 ± 0.03	ND	ND	ND
Triadimenol	ND	ND	ND	ND	ND
Triflumizole	ND	ND	ND	ND	ND
Hexaconazole	0.52 ± 0.03	0.27 ± 0.07	ND	ND	0.49 ± 0.03
Flusilazole	0.19 ± 0.05	ND	ND	ND	0.33 ± 0.01
Diniconazole	ND	ND	ND	ND	ND
Epoxiconazole	ND	ND	ND	ND	ND
Propiconazole	ND	ND	ND	ND	ND
Tebuconazole	ND	ND	ND	ND	ND
Bitertanol	0.20 ± 0.04	ND	ND	ND	ND
Difenoconazole	ND	ND	ND	ND	ND

ND indicates that the content of the sample is less than LOD. Data presented are in means ± standard deviation (*n* = 3).

**Table 4:** Comparison of proposed methods with other methods for determining triazole fungicides.

Adsorbent	Analyte Number	Sample	Determination	LOD (μg/kg)	RSD (%)	Extraction Time (min)	Ref.
CNTs	3	Water	GC-MS	0.02–0.03	<12	>30	[6]
G-Fe_3_O_4_	7	Vegetables	GC-MS	0.01–0.10	<10.6	20	[26]
IL-Fe_3_O_4_@MWCNTs	6	Water	GC-MS	0.05–0.22	<10.5	8	[27]
GCB,C18	5	Medicines	UPLC-MS/MS	0.50–1.10	<11.7	>30	[28]
C/Fe_3_O_4_ NCs	11	Fruits	GC-MS	0.12–0.55	<9.7	2	This work

## Supplementary Materials

The following are available online at http://www.mdpi.com/2079-4991/8/5/302/s1, Table S1: Matrix effect (ME) for 11 triazole fungicides in 4 fruit samples, Table S2: Reusability of the C/Fe_3_O_4_ NCs.
