# Supplementary material for: Carbon-Based Fe3O4 Nanocomposites Derived from Waste Pomelo Peels for Magnetic Solid-Phase Extraction of 11 Triazole Fungicides in Fruit Samples
Source: Nanomaterials (Basel). 2018 May 6;8(5):302. doi: 10.3390/nano8050302 (PMC5977316; doi:10.3390/nano8050302)
Supplement: Supplementary file 1 [file nanomaterials-08-00302-s001.pdf]

Table S1. Matrix effect (ME) for 11 triazole fungicides in 4 fruit samples

Compounds	ME			
	Apple	Pear	Orange	Banana
Triadimefon	0.80	0.87	0/91	0.94
Triadimenol	0.97	0.97	0.97	0.89
Triflumizole	0.94	1.02	1.05	1.09
Hexaconazole	1.09	0.84	0.93	1.07
Flusilazole	0.89	1.09	1.09	1.09
Diniconazole	1.09	0.94	0.93	1.05
Epoxiconazole	0.74	1.08	1.08	1.08
Propiconazole	1.09	0.91	0.91	0.93
Tebuconazole	1.18	0.89	0.89	0.91
Bitertanol	0.89	1.07	1.07	1.10
Difenoconazole	1.04	0.97	1.01	0.99

ME = slope $_{matrix}$/slope $_{solvent}$ [1]

ME: 0.9-1.1, the matrix effect could be ignored

ME: <0.9, matrix weaken effect

ME: >1.1, matrix enhancement effect

[1] Qin, Y. H.; Zhao, P. Y.; Fan, S. F.; Han, Y. T.; Li, Y. J.; Zou, N.; Song, S. Y.; Zhang, Y.; Li; F. B.; Li, X.S.; Pan, C. P. The comparison of dispersive solid phase extraction and multi-plug filtration cleanup method based on multi-walled carbon nanotubes for pesticides multi-residue analysis by liquid chromatography tandem mass spectrometry. *J. Chromatogr. A* 2015, *1385*,1-11.

Table S2. Reusability of the C/Fe_3O_4 NCs

Compounds	Recoveries ± SD (Spiked level: 0.2 mg/L, n=3)			
	Recycle 1	Recycle 5	Recycle 10	Recycle 15
Triadimefon	83.78±1.11a	78.85±1.23b	78.60±1.22b	74.30 ±1.07c
Triadimenol	96.22±0.92a	93.52±0.89a	88.41±1.12a	86.50±1.06b
Triflumizole	73.82±1.56a	72.45±1.28ab	71.91±1.12b	68.53±1.03c
Hexaconazole	88.27±1.07a	86.19±1.21b	84.14±1.32c	79.44±1.23d
Flusilazole	96.41±1.36a	95.54±1.38b	95.12±1.67b	87.43±1.36c
Diniconazole	97.57±1.48a	95.77±1.71b	91.99±1.70c	88.47±1.41d
Epoxiconazole	86.67±1.12a	85.16±1.12b	82.01±1.22c	77.13±1.26d
Propiconazole	96.22±1.66a	93.52±1.65ab	88.41±1.45b	86.50±1.34b
Tebuconazole	88.20±1.14a	86.14±1.37b	85.43±1.24c	81.19±1.35d
Bitertanol	88.38±1.28a	86.65±1.34b	84.79±1.44c	80.43±1.49d
Difenoconazole	89.17±1.18a	86.43±1.26b	84.74±1.39c	80.59±1.34d

The different letters of the same line represent significant differences ($P<0.05$)
